# Sleep Spindles and Fragmented Sleep as Prodromal Markers in a Preclinical Model of LRRK2-G2019S Parkinson's Disease

**DOI:** 10.3389/fneur.2020.00324

**Published:** 2020-05-08

**Authors:** Lindsey M. Crown, Mitchell J. Bartlett, Jean-Paul L. Wiegand, Allison J. Eby, Emily J. Monroe, Kathleen Gies, Luke Wohlford, Matthew J. Fell, Torsten Falk, Stephen L. Cowen

**Affiliations:** ^1^Department of Psychology, University of Arizona, Tucson, AZ, United States; ^2^Department of Neurology, University of Arizona, Tucson, AZ, United States; ^3^Department of Pharmacology, University of Arizona, Tucson, AZ, United States; ^4^Graduate Interdisciplinary Program in Neuroscience, University of Arizona, Tucson, AZ, United States; ^5^Department of Physiology, University of Arizona, Tucson, AZ, United States; ^6^Department of Biomedical Engineering, University of Arizona, Tucson, AZ, United States; ^7^College of Medicine, University of Arizona, Phoenix, AZ, United States; ^8^Merck & Co., Inc., Boston, MA, United States

**Keywords:** Parkinson's disease, prodromal, LRRK2, sleep spindles, sleep fragmentation, EEG, biomarker

## Abstract

Sleep disturbances co-occur with and precede the onset of motor symptoms in Parkinson's disease (PD). We evaluated sleep fragmentation and thalamocortical sleep spindles in mice expressing the p.G2019S mutation of the leucine-rich repeat kinase 2 (*LRRK2*) gene, one of the most common genetic forms of PD. Thalamocortical sleep spindles are oscillatory events that occur during slow-wave sleep that are involved in memory consolidation. We acquired data from electrocorticography, sleep behavioral measures, and a rotarod-based motor enrichment task in 28 *LRRK2*-G2019S knock-in mice and 27 wild-type controls (8–10 month-old males). Sleep was more fragmented in *LRRK2*-G2019S mice; sleep bouts were shorter and more numerous, even though total sleep time was similar to controls. *LRRK2*-G2019S animals expressed more sleep spindles, and individual spindles were longer in duration than in controls. We then chronically administered the LRRK2-inhibitor MLi-2 in-diet to *n* = 12 *LRRK2*-G2019S and *n* = 15 wild-type mice for a within-subject analysis of the effects of kinase inhibition on sleep behavior and physiology. Treatment with MLi-2 did not impact these measures. The data indicate that the *LRRK2*-G2019S mutation could lead to reduced sleep quality and altered sleep spindle physiology. This suggests that sleep spindles in *LRRK2*-G2019S animals could serve as biomarkers for underlying alterations in sleep networks resulting from the *LRRK2*-G2019S mutation, and further evaluation in human *LRRK2*-G2019S carriers is therefore warranted.

## Introduction

Mutations of the leucine-rich repeat kinase-2 (*LRRK2*) gene represent one of the most common genetic causes of Parkinson's disease (PD) (1). As with idiopathic PD, *LRRK2* PD is associated with the progressive loss of dopaminergic neurons in the substantia nigra pars compacta that ultimately results in debilitating motor symptoms such as bradykinesia, rigidity, and tremor (2). *LRRK2*-G2019S is the most prevalent *LRRK2* mutation, accounting for 5–6% of autosomal dominant PD and ~1% of sporadic late-onset PD (3). G2019S is a toxic gain-of-function mutation associated with a variety of cellular effects such as increased glutamatergic activity, neuronal hyper-excitability, deficits in vesicular trafficking, autophagy, and disrupted mitochondrial function (4–6). While work has begun to reveal how the G2019S mutation affects cellular and synaptic function, little is known about how this mutation affects brain circuits.

Although cardinal motor symptoms are most commonly associated with PD, ~80% of patients report sleep problems such as sleep fragmentation, excessive daytime sleepiness, and rapid-eye-movement (REM) sleep behavior disorder (RBD) (7). These symptoms can precede motor symptoms in idiopathic PD by as much as 7 years (8–10). Although sleep disturbances and sleep-associated neurophysiology have been studied in idiopathic PD, much less is known about how sleep is altered in *LRRK2* PD, particularly during the prodromal period. Furthermore, while RBD is one of the earliest prodromal markers of idiopathic PD, it is not as common in *LRRK2* PD (11, 12). Given that sleep disturbances are a feature of *LRRK2* PD (11), there is a need to characterized and identify early sleep alterations unique to *LRRK2* PD, particularly as they relate to non-REM (NREM) sleep.

There are multiple features of the G2019S mutation suggesting that disrupted *LRRK2* expression could alter cellular activity and neural circuits involved in sleep maintenance. For example, *LRRK2* expression is high in the cortex and thalamus (13, 14), two regions involved in the maintenance of NREM sleep. The G2019S mutation is also associated with the potentiation of glutamatergic synapses (6, 15–17), an effect that could excite thalamocortical circuits involved in NREM sleep. One hallmark feature of NREM sleep is the sleep spindle. Sleep spindles are 9–16 Hz thalamocortical oscillations believed to support memory consolidation by coordinating neural activity in cortical, striatal, and limbic circuits (18–20). Spindle density is positively correlated with declarative memory performance, such as the integration of new lexical information (21) and word-pair recall (22). Spindle density is also positively correlated with the refinement of motor skills (23). Given evidence that corticothalamic circuits involved in spindle generation are altered in *LRRK2*-G2019S PD, and evidence for disrupted motor skill learning in PD (24), we hypothesized that the relationship between spindle activity and motor learning would be disrupted in *LRRK2*-G2019S mice.

In this study, we examine the effect of the G2019S mutation on sleep behavior and physiology in *LRRK2*-G2019S knock-in (KI) mice. The G2019S KI mouse is homozygous for the human *LRRK2*-G2019S mutation. Some studies report progressive dopamine-related neurodegeneration and mitochondrial abnormalities by age 12 months but not age 6 months in these mice (25, 26). G2019S KI mice do not reliably display gross motor impairments, though there have been reports of increased exploratory behavior (27), hyperkinesia at 3 months of age (28), and resiliency to social stress (29).

Given the link between sleep disturbances and PD (8–10), we hypothesized that G2019S KI mice would show disrupted sleep patterns relative to wild-type (WT) controls. Specifically, it was hypothesized that G2019S mice would express reduced measures of sleep quality and, given evidence for potentiated glutamatergic transmission with the G2019S mutation, that spindle oscillations would be enhanced.

To investigate these questions, sleep structure, behavior, and spindle oscillations were analyzed in G2019S KI mice and WT controls. Additionally, to determine whether excessive kinase activity altered sleep physiology, the LRRK2-inhibitor MLi-2 was administered to G2019S and WT mice to determine if the drug restored physiological or behavioral effects that resulted from the *LRRK2*-G2019S mutation.

## Methods

### Subjects

A total of *n* = 28 *LRRK2*-G2019S KI (C57BL/6-Lrrk2tm4.1Arte) and *n* = 27 C57BL/6 WT (C57BL/6NTac) control male mice from Taconic Farms (Rensselaer, NY) were acquired between 8 and 16 weeks of age, and aged in the colony room until they reached 8–10 months. Mice were housed in a room with 12-h light/dark cycles, and experiments were performed during the light cycle. Mice had *ad-libitum* access to food and water. All procedures were approved by the Institutional Animal Care and Use Committee at the University of Arizona and conformed to the guidelines of the National Institutes of Health. Two weeks prior to surgery and until experiments began mice were handled for ~15 min a day for 5 days/week. One week before surgery, mice were switched to a control diet [D01060501 from Research Diets Inc. (New Brunswick, NJ)]. Mice were pair-housed until 5 days before surgery after which they were individually housed to avoid damage to implanted electrode arrays. Following experimentation, mice were euthanized with CO_2_ and cardiac puncture. Cortical tissue was collected, flash frozen, and sent to the Fell laboratory for analysis of LRRK2 expression, as in Fell et al. (30).

### Surgical Procedure

**Figure 1:**
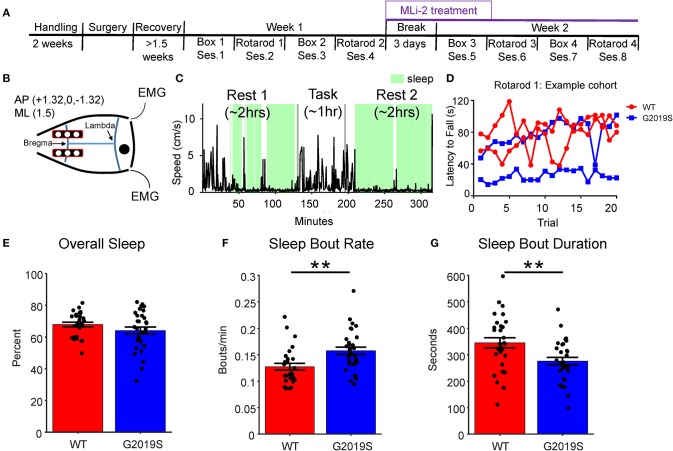
Experimental Overview and Sleep Behavior. **(A)** The experiment took place over 2 weeks. Each week animals alternated between (2x) empty box days and (2x) rotarod days. During the second week a subset of animals were given food containing MLi-2. **(B)** Two rows of three electrodes were placed bilaterally on the cortical surface over M1 and S1 at AP +3.1, 0, −3.1 ML +/−1.5. **(C)** Sleep was identified (shown in green) during Rest 1 and Rest 2 based on movement, inertial data and EMG. **(D)** Example rotarod data obtained from one cohort of mice on Rotarod 1. Each mouse's latency to fall across 20 trials was recorded to obtain a daily average as well as a within-day learning slope. **(E)** G2019S and WT slept a similar amount of time overall as measured by percent time asleep during Rest 1 and Rest 2. **(F)** G2019S animals had more frequent sleep bouts (bouts per minute) than WT mice (t_58_ = −3.201, **p < 0.01). **(G)** G2019S animals had shorter sleep bouts than WT mice (t_54_ = 3.048, **p < 0.01). Error bars indicate ± SEM.

Mice were anesthetized with 3% isoflurane, placed in the stereotactic apparatus, and then given subcutaneous carprofen or ketoprofen (5 mg/kg). Isoflurane levels were subsequently kept between 1 and 2%, the skull was cleaned, and Metabond (Parkell, Edgewood, NY) was applied to the skull surface. Two rectangular craniotomies were drilled bilaterally and centered at AP: 0 mm ML: ± 1.5 mm. Electrocorticography (ECoG) arrays consisting of three 0.4 mm diameter gold pins (Mill-Max Mfg. Corp., Oyster Bay, NY) were placed on the cortical surface in each craniotomy (Figure 1B). A reference gold pin was placed on the cerebellum and two stainless steel electromyography (EMG) wires were inserted into the neck muscle. The electrode arrays were secured to the skull with dental cement. Mice were allowed 10–12 days to recover before regular recording sessions began. Five days prior to the first recording session, the quality of the ECoG signals were checked and each mouse was exposed to their sleep box for 10 min, the rotarod training apparatus (Rotarod task) for 2 min, and an empty box (Box task) for 5 min in order to reduce novelty effects.

### Data Acquisition

Neural, EMG, and inertial data were acquired using the Intan data acquisition system (Intan Technologies Inc., Los Angeles, CA). ECoG and EMG signals were acquired at 12.5 kHz. Overhead position tracking data was gathered at 30 frames-per second by a Manta GigE camera (Allied Vision, Exton, PA). Between 2 and 4 mice were recorded simultaneously from mixed genotype groups.

### Sleep and Box Recording Days

During neural recordings of sleep, animals were housed in 18 x 18 cm polycarbonate boxes. Each box was enclosed in a metal mesh Faraday cage and sound-attenuating foam. Each sleep box contained bedding from the mouse's home cage. Recording sessions occurred 4 days a week for 2 weeks. Each session had the same structure (Figures 1A,C) whereby mice were plugged into the recording apparatus approximately 3 h into the beginning of their light cycle. The recording session consisted of 2 h of pre-task sleep (Rest 1) in the sleep box followed by a 1-h task condition which involved either the exploration of an empty box (Box) or the rotarod motor training task (Rotarod). Completion of the task was followed by a second 2-h sleep period (Rest 2). The task (Box or Rotarod) was switched on alternating days (Figure 1A). In the Box task, mice were placed in a clean and empty polycarbonate box and left undisturbed for 1 h. In the Rotarod task, the mice were unplugged from the recording apparatus and placed on the rotarod. The details of the Rotarod task are described below.

### Rotarod Training Task

This task was based on the motor-learning paradigm described in Li et al. (31). The rotarod apparatus had 4 lanes, 1 per mouse. Mice were placed on the rod and allowed to rest for 1 min after which the rod began to rotate and accelerated from 0 to 79 rpm over 3 min. Once the last mouse fell off the rod, the rod was stopped, and mice were placed back on the rod in the order they fell off. This process was repeated for 20 trials (Figure 1D). All mice were video recorded and latency to fall was subsequently scored by a research assistant blinded to genotype.

### Drug Administration

Following the 4th recording session (Week 1), mice either continued to have *ad-libitum* access to control chow (Research Diets D01060501, 10% kcal fat and cornstarch) or *ad-libitum* access to chow containing LRRK2 inhibitor MLi-2. MLi-2 was added as powder by Research Diets and chow was otherwise identical to control. MLi-2 chow was formulated to provide concentrations of 60 mg/kg (30). In a study of chronic in-diet administration of MLi-2 over 11 days, this dosage has been shown to reduce the ratio of pS935 to total LRRK2 to <0.1 after 4 h (30).

Drug group assignment was random, and experimenters were blind to drug condition. Each recording cohort contained at least one mouse on MLi-2. Mouse weight and food intake was recorded daily. Mice remained on MLi-2 chow until the conclusion of experiment and were euthanized after a total of 3 weeks MLi-2 exposure. Phosphorylation of residue S395 of the LRRK2 protein was used as a read-out of LRRK2 kinase activity and thus following euthanasia, extent of kinase inhibition was assessed by analysis of pSer935 LRRK2/total LRRK2 in cortex by western blot, as described prior (30). Animals identified as having insufficient kinase inhibition (*n* = 7 WT and *n* = 2 G2019S) on drug or inappropriately low kinase activity (*n* = 1 G2019S) on vehicle were removed from analysis of drug-related effects. In animals used for analysis of MLi-2-related effects, there was > 90% reduction of kinase activity after treatment evident for both WT and transgenic mice (Supplementary Figure 1).

### Analysis

#### Signal Processing and Statistical Analyses

ECoG signals were analyzed using Fourier and wavelet measures of spectral power and frequency using custom Matlab™ functions. Normality of distributions were checked with the Anderson-Darling test. The Wilcoxon Rank-Sum test was used for non-parametric data. The Holm multiple comparisons correction was used for *post-hoc* comparisons. Cohen's d was used as a measure of effect size.

#### Inertial Measurement and EMG

Inertial data was obtained through a sensor mounted on the neural recording headstage. Inertial data has been demonstrated to provide an excellent readout of sleep/wake state (32). Motion was quantified by summing the absolute value of the first derivative of acceleration (“jerk” or |m/s^3^|). A threshold of 1 m/s^3^ was set for all datasets based on visual inspection. EMG signals were band-pass filtered (70–250 Hz) and the absolute value of the signal was smoothed using a 200 ms moving average to measure muscle tone (33).

#### Identification of Sleep

To be classified as sleep, two of three conditions had to be met: (1) inertial data <1 m/s^3^, (2) EMG activity < a session-by session visually scored threshold, and (3) speed <2 cm/s. If two of these conditions were true for > 40 s (34), the period was classified as sleep. Analyses of sleep behavior was restricted to 110-min periods beginning 5 min after the start of each Rest epoch in order to eliminate possible artifact from researcher presence at the start and end of the session.

### Identification of Sleep-Spindles

Spindles were identified using a threshold-crossing approach similar to Phillips et al. (35). The analysis of spindles involved *n* = 22 G2019S and *n* = 26 WT animals. Animals were excluded if (1) data from only one hemisphere was acquired, (2) the animal did not complete the experiment, or (3) no spindles were identified on >2 days during Week 1 or >2 days during Week 2. To reduce common noise and identify local spindle events, common average re-referencing was implemented (36). A common average of the left hemisphere electrodes was subtracted from the signal of the right anterior electrode. This electrode was used for spindle identification due to its proximity to motor cortex (M1) and distance from potential hippocampal REM sleep-associated theta volume conduction.

To identify spindles, the ECoG signal was bandpass filtered to the sigma band (9–16 Hz, 12th order Butterworth filter) and smoothed with a 20-ms Hanning window. A threshold was set by calculating a trimmed (Winsorized) standard deviation (between 10 and 90th percentiles) based on sigma power during sleep. Candidate spindle events were identified when sigma power was >2.5 standard deviations and remained above 1.7 standard deviations for ≥500 ms and ≤ 2 s. Only events that occurred during identified sleep were included. The oscillatory frequency of each spindle was determined using Burg's method (40th order; pburg Matlab function).

#### Measuring the Relationship Between Spindle Activity and Behavioral Performance

Given the role of sleep spindles in memory consolidation, we evaluated the relationship between motor performance and learning with post-task spindle density. The relationship between motor learning and spindle density was measured by measuring the Pearson's correlation coefficient between the change in spindle density from Rest 1 to Rest 2 and the mean latency to fall. This was only performed on Rotarod 1 and Rotarod 2, both off-drug days for which there was within-day rotarod learning as determined by a significant positive correlation (*p* < 0.05) between trial number and the latency to fall.

## Results

### LRRK2-G2019S Mice Expressed Fragmented Sleep

Sleep fragmentation was assessed as the number of sleep bouts per minute and the average sleep bout duration. Data from Rest 1 and Rest 2 were combined, and only Week 1 data was used in this analysis in order to identify genotypic differences in sleep quality. We observed that G2019S KI mice slept a similar amount of time as WT mice (t_54_ = 1.656, *p* = 0.104; Figure 1E); however, G2019S mice had significantly more sleep bouts (t_58_ = −3.201, *p* = 0.002, *d* = 0.825; Figure 1F), and these sleep bouts were shorter in duration than WT mice (t_54_ = 3.048, *p* = 0.004, *d* = 0.782; Figure 1G). To assess the effect of task on sleep quality measures, Rest 2 sleep features following Rotarod and Box tasks were compared for WT and G2019S animals. WT but not G2019S animals slept more following Rotarod compared to Box (t_28_ = 3.251, *p* = 0.006 for WT, *d* = 0.604; Supplementary Figure 2A). Both groups had a greater sleep bout rate following Rotarod sessions (t_29_ = 3.25, *p* = 0.006, *d* = 0.605 for WT; t_27_ = 2.192, *p* = 0.037, *d* = 0.534 for G2019S; Supplementary Figure 2B) and there was no effect of task on sleep bout duration for either group (t_27_ = −0.014, *p* = 0.989 for WT; t_28_ = −2.015, *p* = 0.107 for G2019S; Supplementary Figure 2C).

### Sleep Spindle Density and Duration Are Increased in LRRK2-G2019S Mice

**Figure 2:**
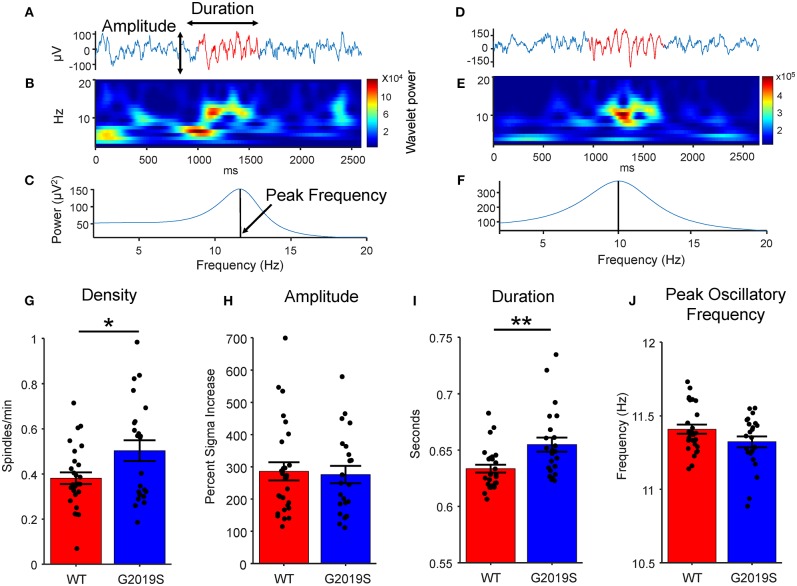
Spindle Identification and Properties. Two example traces with common average re-referenced signal show in blue and identified putative spindle shown in red, **(A)** one from a WT mouse and **(D)** one from a G2019S mouse. **(B,E)** Wavelet spectrogram of putative spindle events. **(C,F)** Spindle power spectral density using Matlab's pburg function. **(G)** G2019S animals had a greater sleep spindle density (spindles/minute of sleep) than did WT animals (t_40_ = −2.17, ^*^p < 0.05). **(H)** There was no difference in spindle amplitude by genotype. **(I)** G2019S animals had significantly longer duration spindles than WT controls (t_49_ = −2.862, ^**^p < 0.01 Wilcoxon Rank-Sum). **(J)** There was no significant difference between WT and G2019S peak spindle oscillatory frequency. Error bars indicate ± SEM.

Evidence for enhanced synaptic excitability in the *LRRK2*-G2019S mutation (6, 15–17) led to the hypothesis that increased cortical glutamatergic output would result in increased sleep spindle density. Example candidate spindle events, wavelet spectrograms and spindle power spectral densities are shown in Figures 2A–F. Accordingly, spindle density in G2019S animals during Week 1 (pre-drug) was greater than WT animals (t_40_= −2.17, *p* = 0.036, *d* = 0.604; Figure 2G). We chose the rotarod motor learning task to induce spindles in post-task sleep (Rest 2). While Rest 2 showed a greater spindle density than Rest 1 for nearly all mice, surprisingly we found that spindle density was greater following the Box task compared to the Rotarod task. This was true both as a relative change in spindle density from Rest 1 to Rest 2 (t_24_ = −4.131, *p* = 0.005, *d* = 0.647 in WT; t_22_ = −3.104, *p* = 1.64 × 10^−4^, *d* = 0.825 in G2019S; Supplementary Figure 3A) and by comparing spindle density in Rest 2 alone, for which G2019S animals shows a greater Box relative to Rotarod spindle density difference (t_46_ = −8.543, *p* = 4.76 × 10^−11^, *d* = −2.43, Supplementary Figure 3B).

We also hypothesized that enhanced synaptic excitability in mice carrying the G2019S mutation would lead to higher spindle power, frequency, and duration. Spindle amplitude was quantified as the percent increase in sigma power during a spindle from baseline sleep. No difference in peak spindle frequency (t_49_ = 1.927, *p* = 0.060; Figure 2J) or amplitude (t_49_ = 0.507, *p* = 0.614; Figure 2H) was observed between G2019S and WT mice. Spindle durations were significantly longer in G2019S mice (t_49_ = −2.862, *p* = 0.007, *d* = 0.802; Figure 2I). Rotarod performance did not differ between WT and G2019S animals.

### Rotarod Performance Did Not Differ Between WT and LRRK2-G2019S Animals

**Figure 3:**
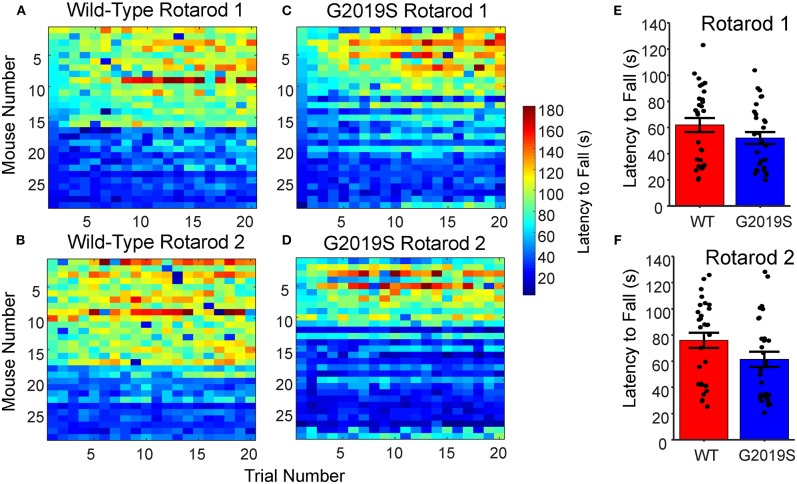
Rotarod Motor Learning Task. **(A–D)** Raw latency to fall measures for all WT and G2019S mice sorted by performance for Rotarod 1 and 2. **(E,F)** No significant differences were present for average latency to fall between G2019S and WT for either Rotarod 1 or Rotarod 2 (*t*-test, *p* > 0.05). Error bars indicate ± SEM.

**Figure 4:**
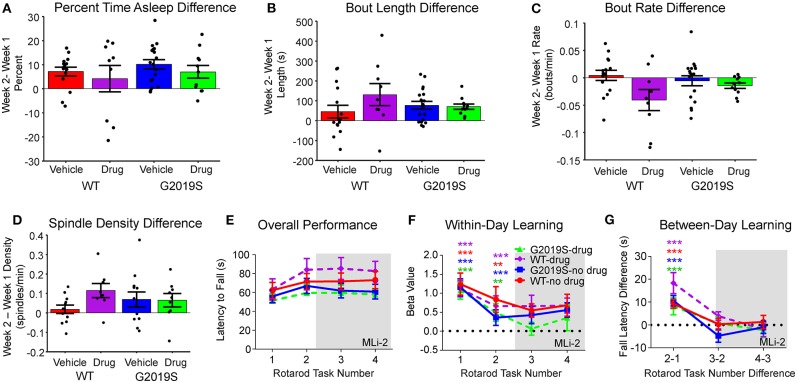
Effect of MLi-2. There was no effect of MLi-2 on **(A)** percent time asleep, **(B)** sleep bout length, or **(C)** sleep bout rate as measured by the within-animal difference of Week 2- Week 1. **(D)** Similarly, MLi-2 had no effect on spindle density measures. **(E)** Rotarod Performance did not change with MLi-2 administration, nor were there group differences in **(F)** within-day learning or **(G)** between-day learning by drug condition.

Rotarod performance and learning was analyzed for WT and G2019S mice during Week 1 to assess genotypic differences. The mean latency to fall across all 20 trials of the Rotarod task was used to measure overall motor performance for each mouse (Figures 3A–D). The mean latency to fall did not differ significantly between G2019S and WT animals (Wilcoxon-Rank Sum, *p* = 0.099; Figures 3E,F). Regressing trial number against latency to fall revealed within-day learning for both WT and G2019S animals on Rotarod 1 (t_28_ = 6.75, *p* = 2.53 x 10^-7^ for WT and t_28_ = 7.10, *p* = 9.94 x 10^-8^ for G2019S; Figure 4E) and Rotarod 2 (t_28_ = 3.55, *p* = 0.001 for WT and t_28_ = 2.73, *p* = 0.011 for G2019S; Figure 4F). Both groups also showed an increased mean latency to fall from Rotarod 1 to Rotarod 2 (t_28_ = 5.26, *p* = 1.37 x 10^-5^ for WT and t_28_ = 4.60, *p* = 8.39 x 10^-5^ for G2019S; Figure 4G). There were no genotypic differences in these learning metrics (t_52_ = 0.88, *p* = 0.383).

To determine if spindle activity was related to within-session learning, we also regressed learning slopes with changes in spindle density from Rest 1 to Rest 2. This analysis did not identify any within-day effect for Rotarod 1 (*R* = −0.166, *p* = 0.462 in WT; *R* = 0.055, *p* = 0.813 in G2019S; Supplementary Figure 4A) or Rotarod 2 (*R* = 0.340, *p* = 0.122 in WT; *R* = −0.202, *p* = 0.381 in G2019S; Supplementary Figure 4B). The same analysis was done on measures of distance traveled in the Box task (*R* = −0.068, *p* = 0.742 for WT; *R* = −0.063, *p* = 0.793 for G2019S; Supplementary Figure 4C) and between-day learning with no significant correlations observed (*R* = 0.297, *p* = 0.179 in WT; *R* = 0.336, *p* = 0.137 in G2019S; Supplementary Figures C,D).

### 4–7 Day in-Diet Treatment of 60 mg/kg LRRK2 Inhibitor MLi-2 Did Not Alter Sleep Behavior, Physiology, or Rotarod Performance

The above analyses were repeated as a within-subject comparison (Week 2 - Week 1) of behavior and physiology for WT-vehicle, WT-drug, G2019S-vehicle, and G2019S-drug. No changes in percentage of time asleep (F_3,49_ = 0.784, *p* = 0.508; Figure 4A), mean sleep bout length (F_3,49_ = 1.181, *p* = 0.327; Figure 4B), or mean sleep bout rate (F_3,49_ = 2.724, *p* = 0.054; Figure 4C) were observed. Similarly, there were no group differences in rotarod performance during Week 2 with drug administration (F_3,45_ = 2.71, *p* = 0.056; Figures 2C–E).

It was hypothesized that if the observed increase in sleep spindle density in G2019S mice (Figure 3J) was the result of excessive kinase activity, MLi-2 should reduce spindle density in G2019S mice during Week 2. Subtracting Week 2 average spindle density from Week 1 average spindle density, we tested the hypothesis that G2019S-drug animals would show decreased Week 2 spindle density, but observed no effect (F_3,36_ = 1.300, *p* = 0.290; Figure 4D).

## Discussion

Sleep disruption is strongly associated with PD, and impaired sleep often precedes the onset of motor symptoms (9, 37, 38). Despite the close relationship between sleep disruption and PD, few studies have looked at how the G2019S mutation affects sleep behavior, and none to our knowledge have examined how sleep is altered in *LRRK2*-G2019S KI mice. Consistent with reports of sleep disturbances in *LRRK2* PD patients (11), we observed disrupted sleep in *LRRK2*-G2019S mice. Specifically, while total sleep time in G2019S and WT mice was similar, sleep bouts in G2019S mice were shorter and more frequent, indicating sleep fragmentation. These effects were not rescued by delivery of MLi-2, a potent LRRK2 inhibitor. In addition, sleep spindles were longer and more frequent in G2019S mice.

While no previous studies to our knowledge have examined sleep fragmentation in G2019S mice, sleep fragmentation (39) and insomnia (40) have been identified in other animal models of PD (41), and are reported in patients with idiopathic (38, 42) and *LRRK2* PD (11, 43). We found that G2019S mice expressed fragmented sleep (Figure 1F), adding validity to the *LRRK2*-G2019S KI model and suggesting that *LRRK2*-G2019S animals show prodromal PD symptoms.

A recurring concern for the study of *LRRK2* PD in mice has been the difficulty identifying motor deficits (5). Accordingly, we found no evidence for motor impairment in 8–10 month old *LRRK2*-G2019S mice. It is possible that the rotarod may have not be well-suited to identify gross motor deficits, as one study found that *LRRK2*-G2019S animals showed decreased performance on bar and drag tests at 6 months, but not the rotarod (28).

Our study is the first to identify physiological changes in sleep in the *LRRK2*-G2019S KI mouse model. Specifically, sleep-spindle density and duration were increased in *LRRK2*-G2019S mice. *LRRK2* is expressed in the thalamus and cortex, two structures crucial for the generation and maintenance of spindle oscillations (13, 14). Increased LRRK2 kinase activity resulting from the G2019S mutation has been shown to enhance neuronal excitability and glutamate release in cortical cells from *LRRK2*-G2019S KI mice (6, 44). Increased excitability could stimulate corticothalamic circuits, resulting in increased spindle density and duration. While we also predicted the oscillatory frequency and amplitude of spindles would be enhanced, no effect on these features was identified.

We observed that suppression of kinase activity through in-diet administration of MLi-2 did not alter sleep fragmentation, spindle density, or spindle duration. This suggests that the immediate effects of the G2019S mutation did not drive the observed effect on spindle activity. It is therefore possible that long-term developmental effects from persistent increased LRRK2 activity contributed to altered spindle activity and sleep quality in G2019S mice. Furthermore, because MLi-2 was only administered for 1 week, the lack of any observed effect of MLi-2 on sleep is not necessarily indicative for the effects of long-term treatment with MLi-2.

While we observed increased spindle density in G2019S mice, there is evidence that patients with idiopathic PD express fewer spindles relative to healthy controls (38). Therefore, *LRRK2* PD may differ from idiopathic PD in its effect on spindle oscillations. Differences in sleep physiology between idiopathic and *LRRK2* PD are also suggested by the observation that while RBD is a common feature of idiopathic PD, it is less common in *LRRK2* PD (45). Future studies could test relationship between spindle density and *LRRK2*-G2019S in human *LRRK2*-G2019S patients using polysomnography.

In summary, the results of the present study suggest a link between the *LRRK2*-G2019S mutation and alterations in behavioral and physiological features of sleep in mice. None of these changes were affected by 4–7 day in-diet suppression of LRRK2 activity via MLi-2, suggesting that neural circuit and developmental changes induced by the G2019S mutation extend beyond increased kinase activity. Furthermore, the identification of increased sleep fragmentation, increased sleep spindle density, and longer sleep spindle duration may serve as early biomarkers of *LRRK2* PD.

## Data Availability Statement

The datasets generated for this study are available on request to the corresponding author.

## Ethics Statement

The animal study was reviewed and approved by Institutional Animal Care and Use Committee at the University of Arizona (IACUC).

## Author Contributions

LC designed the study, collected the data, performed the analysis, and wrote the manuscript. MB designed the study, collected the data and assisted with review. J-PW designed the study and performed the analysis. AE collected the data and performed the analysis. EM collected the data and performed the analysis. KG collected the data. LW collected the data and performed the analysis. MF performed the histological analysis. TF designed the study and edited and reviewed the manuscript. SC designed the study, performed the analysis, and wrote the manuscript.

## Conflict of Interest

MF is a paid employee of Merck & Co., Inc., Boston, MA, United States. The remaining authors declare that the research was conducted in the absence of any commercial or financial relationships that could be construed as a potential conflict of interest.
