# Supplementary material for: Sleep Spindles and Fragmented Sleep as Prodromal Markers in a Preclinical Model of LRRK2-G2019S Parkinson's Disease
Source: Front Neurol. 2020 May 8;11:324. doi: 10.3389/fneur.2020.00324 (PMC7232828; doi:10.3389/fneur.2020.00324)
Supplement: Supplementary file 1 [file Data_Sheet_1.DOCX]

**Supplemental Information**


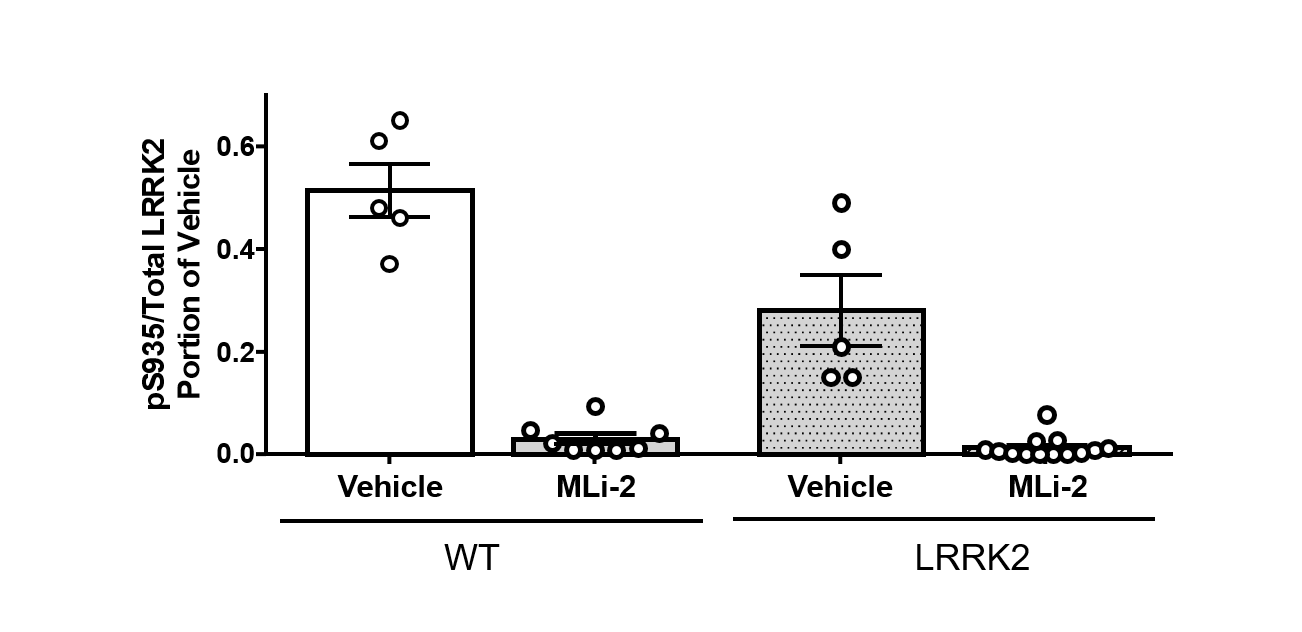


**Supplementary Figure 1.** Treatment with MLi-2 reduced the proportion of pS935/Total LRRK2 in both WT and LRRK2 animals (F_1,37=_12.08, *p=*0.001). Animals that did not show sufficient kinase inhibition (n=7 WT n=2 LRRK2) or sufficiently high pS935/Total LRRK2 levels for vehicle (n=1 LRRK2) were removed from analysis of Week 2 drug-related effects.

## Sleep bout rate increased following rotarod training.

To examine the effect of recent motor learning on sleep quality, the percent time asleep, sleep bout rate, and sleep bout duration following the task (Rest 2 Rotarod or Box) were compared to determine if animals slept more or in a less-fragmented manner following a learning task. **Supplementary Figure 2** presents the three sleep features assessed during the post-task Rest 2 epoch. Each point indicates the value for a given mouse under the Box (x-axis) or Rotarod (y-axis) conditions. Statistical comparisons were performed using a within-subject measure of the difference between the empty box and rotarod conditions (e.g., Percent time slept during Rest 2 Rotarod – Percent time slept during Rest 2 Box).

WT animals but not LRRK2-G2019S mice slept more following Rotarod relative to Box (t_28_=3.251, *p*=0.006 for WT, d=0.605; t_29_=0.671, *p*=0.508 for LRRK2; Holm-corrected for 2 comparisons; **Supplementary Figure** **2A**), but there was no significant difference between groups (t_57=_-1.332, *p*=0.190). In addition, both LRRK2 and WT animals had a higher sleep-bout rate following rotarod sessions (t_29_=3.25, *p*=0.006, d=0.605 for WT; t_27_=2.192, *p*=0.037, d=0.534 for LRRK2; Holm-corrected, 2 comparisons; **Supplementary Figure** **2B**), and there were also no group differences (t_56_=0.926, *p* =0.359). Although mean bout durations were significantly longer in WT mice (**Supplementary Figure** **2C**), the relative difference of bout duration following Rotarod and Box was not different in either group (t_27_=-0.014, *p*=0.989 for WT, t_28_=-2.015, *p*=0.107 for LRRK2; **Supplementary Figure** **2C**).


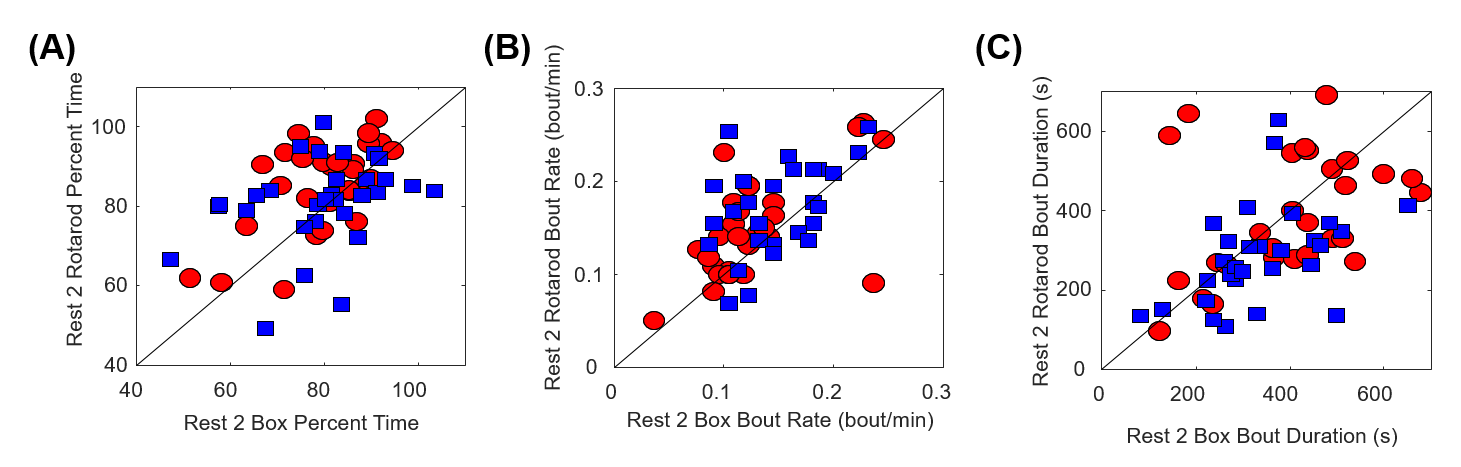


**Supplementary Figure 2.** Effect of Task on Sleep Behavior Measures. **(A)** Comparing Rotarod and Box days, there was no difference in percent time sleeping for LRRK2-G2019S animals but WT animals slept more after Rotarod than Box sessions (t_28_=3.251, *p* < 0.01 Holm corrected for multiple comparisons). **(B)** LRRK2-G2019S and WT mice had more frequent sleep bouts following Rotarod sessions than Box sessions as measured by Rate_Rotarod_ – Rate_Box_ (t_29_=3.25, *p* < 0.05 for WT, t_27_=2.192, *p*<0.01 for LRRK2, Holm corrected for multiple comparisons). **(C)** There was no effect of task on sleep bout duration for either LRRK2-G2019S or WT mice.

***Spindle density increased more following Box than Rotarod sessions.***

We hypothesized that spindle density would increase following Rotarod relative to Box sessions in response to training-induced memory consolidation. Contrary to this prediction, the changes in spindle density from Rest 1 to Rest 2 was greatest following the Box task for both LRRK2-G2019S and WT animals (t_24_=-4.131, *p*=0.005, d=0.647 in WT; t_22_=-3.104, *p=*1.64x10^-4^, d=0.825 in LRRK2; **Supplementary** **Figure 3A**), and no between-group differences (t_46_=-0.425, *p=*0.673) were observed. However, when comparing Rest 2 sleep alone, we found that LRRK2-G2019S animals displayed this effect more than WT (t_46_=-8.543, *p*=4.76x10^-11^, d=-2.43; **Supplementary** **Figure 3B)**


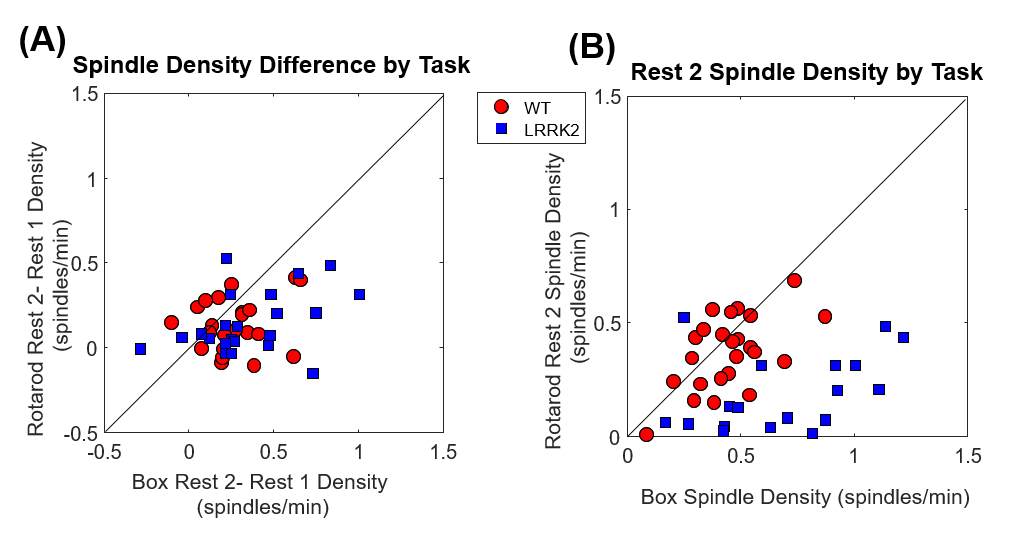


**Supplementary Figure 3.** Effect of Task on Sleep Spindle Density in Post-Task Sleep. **(A)** Comparing the relative increase in spindle density from Rest 1 to Rest 2, both WT and LRRK2 mice showed increased spindle density following Box sessions (t_24_=-4.131, *p*=0.005, d=0.647 in WT; t_22_=-3.104, 1.64x10^-4^, d=0.825 in LRRK2), with no genotypic differences. The diagonal line represents equal spindle density after both conditions. **(B)** Looking only at Rest 2, both WT (t_24_= -3.17, *p*=0.004 d=-0.634) and LRRK2 (t_22_=-10.16, *p*=9.11x10^-10^, d=-2.12) mice had a greater spindle density following Box and LRRK2 showed a significantly greater effect of task on spindle density than WT (t_46_=-8.543, *p*=4.76x10^-11^, d=-2.43).

## Spindle density increase did not correlate with within-day learning, between-day learning, or exploration within the empty box

Because spindles are associated with memory consolidation, we hypothesized that Rest 1 to Rest 2 spindle density increase would be correlated with measures of within or between-day learning. Neither within-day learning, measured by the beta value obtained from a regression of latency to fall values across a session’s 20 trials (**Supplementary Figure** **4A-B**), nor the difference in mean latency to fall from one session to another (**Supplementary Figure** **4D**). were significantly correlated with Rest 2- Rest 1 spindle density increase. We also hypothesized that if the Box task was sufficiently novel then distance traveled within the box could be used as a metric for learning. Correlating this with Rest 2 - Rest 1 spindle density did not yield significant results (**Supplementary Figure 4C**).


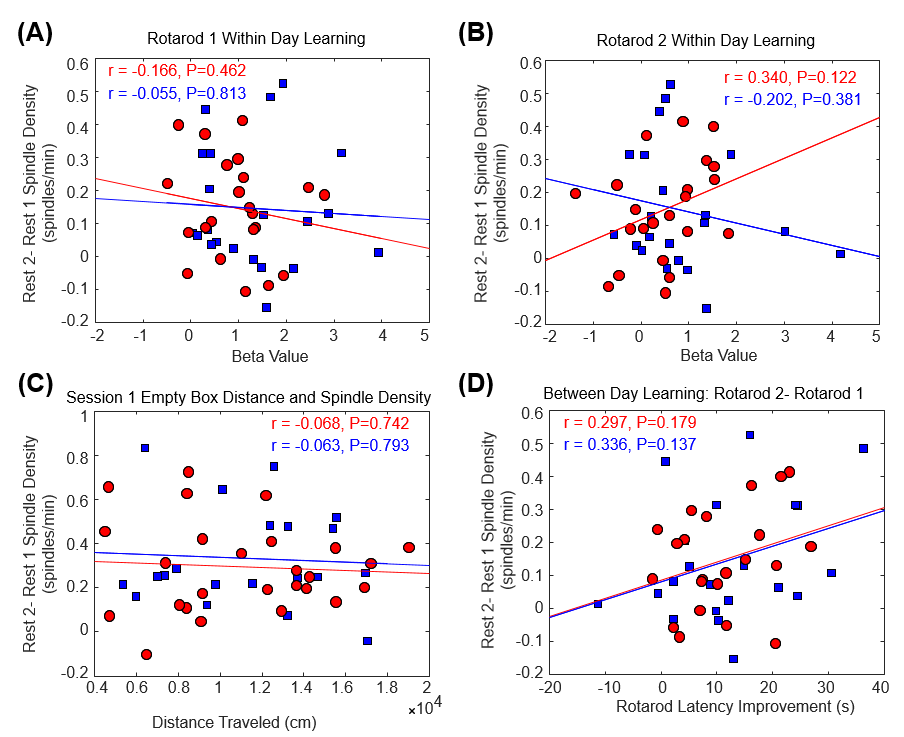


**Supplementary Figure 4.** Spindle Correlations. **(A)** Spindle density increase does not correlate with within-day learning measures for Rotarod 1 or **(B)** for Rotarod 2. **(C)** Spindle density increase does not correlate with distance travelled during the Box task for Box 1 in WT or LRRK2-G2019S. **(D)** Spindle density increase does not correlate with between-day learning, measured as latency-to-fall improvement from Rotarod 1 to Rotarod 2.
